# Preoperative Prediction of Difficult Laparoscopic Cholecystectomy Using a Scoring System: A Prospective Cohort Study

**DOI:** 10.7759/cureus.102055

**Published:** 2026-01-22

**Authors:** Avinash Kumar, Maninder K Chhabra, Puneet Chhibber

**Affiliations:** 1 General Surgery, Deen Dayal Upadhyay Hospital, New Delhi, IND

**Keywords:** difficult cholecystectomy, gallstones, laparoscopic cholecystectomy, predictive score, preoperative risk stratification

## Abstract

Background: Laparoscopic cholecystectomy (LC) is now considered the gold standard for the treatment of symptomatic cholelithiasis. However, conversion to open surgery is occasionally required due to intraoperative difficulties and complications. Early preoperative identification of difficult LC cases can be of help in surgical preparedness and patient counselling.

Objective: To evaluate the sensitivity, specificity, and predictive value of a preoperative scoring system in the prediction of difficult LC.

Methods: A prospective cohort study was conducted on 150 patients undergoing elective LC at Deen Dayal Upadhyay Hospital, New Delhi, India. Each patient was scored preoperatively based on age, sex, history of hospitalization, BMI, abdominal scars, palpable gallbladder, gallbladder wall thickness, pericholecystic fluid, and impacted stones based on the scoring system proposed by Randhawa and Pujahari. Intraoperative findings were recorded to classify cases as easy, difficult, or very difficult based on parameters that include time of surgery, bile/stone spillage, duct/artery injury, conversion to open surgery, difficulties in port access, Calot’s triangle dissection, and gall bladder extraction. Statistical analysis included sensitivity, specificity, predictive values of the preoperative score, and logistic regression analysis.

Results: The mean preoperative score was 2.55 ± 2.55. Intraoperatively, 72% of cases were classified as easy, 24.7% as difficult, and 3.3% as very difficult, with a conversion rate to open surgery of 3.3%. Thickened gallbladder wall (>4 mm), presence of pericholecystic fluid, impacted stones, and a BMI >27.5 were found to be significantly associated with increased operative difficulty (p < 0.05). The scoring system demonstrated a sensitivity of 57.14% and a specificity of 79.51%, while the positive predictive value and negative predictive value were 39% and 89%, respectively.

Conclusion: The proposed scoring system is a reliable, simple tool for predicting difficult LC preoperatively. Incorporation of this tool can assist in preoperative planning and improve surgical outcomes by better preparing and minimizing unexpected conversions.

## Introduction

Gallstone disease is one of the most prevalent hepatobiliary disorders worldwide, affecting approximately 11-36% of the population. Although the majority of individuals with gallstones remain asymptomatic, nearly 3% of asymptomatic patients develop symptoms annually [1]. In India, gallstone disease affects around 10-20% of the population, with a higher incidence observed among females.

Laparoscopic cholecystectomy has become the standard treatment for cholelithiasis [2] owing to its well-documented benefits, including reduced pain in the postoperative period, reduced hospital stay, better recovery, and improved cosmetic outcomes when compared to open cholecystectomy [3]. Since the first laparoscopic cholecystectomy was performed in 1985 [1], minimally invasive techniques have rapidly evolved, progressing from conventional multiport approaches to single-incision and robot-assisted procedures [4].

Despite advancements in surgical expertise and technology, laparoscopic cholecystectomy can still be technically demanding in a subset of patients. Difficult laparoscopic cholecystectomy is commonly characterized by prolonged operative time, increased technical complexity, intraoperative complications, the need for partial cholecystectomy, or conversion to open surgery. Reported conversion rates range between 3% and 12% globally [4,5].

Several patient-related, clinical, and radiological factors have been implicated in increasing operative difficulty, including advanced age, male sex, obesity, previous upper abdominal surgery, gallbladder wall thickening, pericholecystic fluid, impacted stones, and acute or chronic inflammatory changes [4]. Intraoperative challenges, such as dense adhesions in Calot’s triangle, distorted anatomy, uncontrolled bleeding, and gallbladder perforation, further contribute to operative difficulty and postoperative morbidity, such as bilioma, which is a deadly complication [6].

Conversion to open cholecystectomy is associated with increased postoperative pain, longer hospitalization, higher risk of wound and respiratory infections, and increased healthcare costs. Therefore, the ability to anticipate difficult laparoscopic cholecystectomy before surgery is of considerable clinical importance, as it allows for better surgical planning, appropriate patient counselling, optimized operating room scheduling, and timely referral to experienced hepatobiliary surgeons when necessary [6,7].

Various predictive models and scoring systems have been proposed to estimate the difficulty of laparoscopic cholecystectomy, among which the scoring system described by Randhawa and Pujahari is widely recognized. The present study addresses the unmet need of a lack of prospective validation in the local population. The present study was designed to evaluate a modified preoperative scoring system based on clinical history, physical examination, and ultrasonographic findings and to assess its effectiveness in predicting difficult laparoscopic cholecystectomy in a prospective cohort [8].

## Materials and methods

Study design and setting

This was a prospective cohort study conducted at Deen Dayal Upadhyay Hospital, New Delhi, India, over a period of 18 months from February 2021 to July 2022. The study included 150 patients who underwent laparoscopic cholecystectomy during the study period. The study was approved by the Institutional Ethical Committee.

Sample size calculation

The sample size calculation was based on the study by Gupta et al. [9], who used different preoperative variables to predict the difficult laparoscopic cholecystectomy based on history, clinical examination, and sonological findings and then assessed the intraoperative findings to correlate both.

The study of Gupta et al. observed that the sensitivity and specificity of the scoring system to predict difficult laparoscopic cholecystectomy was 95.74% and 73.68%, respectively. Taking these values as reference, the minimum required sample size with desired precision of 10%, 80% power of the study, and 5% level of significance is 138 patients based on the specificity of the test and 48 based on the sensitivity of the test; to reduce the margin of error, the total sample size taken is 150. Consecutive sampling was used to minimize selection bias.

Study population

Adult patients aged 18 years and above who were scheduled for elective laparoscopic cholecystectomy for cholelithiasis were included in the study after getting a written informed consent from every patient. Patients undergoing combined surgical procedures were excluded; all patients with acute cholecystitis, GB malignancy, pancreatitis, or common bile duct (CBD) stones were excluded from the study. All the patients undergoing emergency cholecystectomy were excluded from the study.

Preoperative assessment

All patients underwent thorough preoperative assessment, which included detailed clinical history, general and systemic examination, ultrasonography of the abdomen, and routine laboratory investigations. Patients who met the inclusion criteria were evaluated using a preoperative scoring system.

Preoperative scoring system

The preoperative scoring system used in the study was previously validated by Randhawa and Pujahari in their study in 2009 [8]. The preoperative scoring system was based on the parameters derived from history, clinical examination, and ultrasonographic findings. Historical parameters included age, sex, and history of hospitalization for cholecystitis. Clinical parameters included body mass index, presence of an abdominal scar, and palpable gallbladder. Ultrasonographic parameters included gallbladder wall thickness greater than 4 mm, presence of pericholecystic collection, and presence of an impacted stone. Based on the scores obtained, patients were categorized as having easy, difficult, or very difficult laparoscopic cholecystectomy. The details of the scoring system are summarized in Table 1.

**Table 1:** Preoperative parameters of the scoring system with their specific scores and maximum possible scores BMI: Body mass index

Parameter Category	Variable	Criteria	Score	Maximum Score
History	Age	<50 years	0	1
		>50 years	1	
	Sex	Female	0	1
		Male	1	
	History of hospitalization	No	0	1
		Yes	1	
Clinical	Body mass index (BMI)	<25 kg/m²	0	2
		25–27.5 kg/m²	1	
		>27.5 kg/m²	2	
	Palpable gallbladder	No	0	1
		Yes	1	
	Abdominal scar	No	0	2
		Infraumbilical	1	
		Supraumbilical	2	
Ultrasonography	Gallbladder wall thickness	Thin	0	2
		>4 mm	2	
	Impacted stones	No	0	1
		Yes	1	
	Pericholecystic collection	No	0	1
		Yes	1	

Surgical procedure and intraoperative assessment

All patients underwent elective laparoscopic cholecystectomy using the standard four-port technique. During surgery, the operating surgeon assessed the operative difficulty based on factors such as difficulty in umbilical port entry, difficulty in gallbladder grasping, difficulty in adhesiolysis, difficulty in dissection of Calot's triangle, difficulty in clipping of the cystic duct, difficulty in clipping of the cystic artery, and difficulty in extraction of the gallbladder. Additional intraoperative assessment criteria included duration of surgery, bile or stone spillage, duct or artery injury, and conversion to open surgery. These intraoperative objective assessment criteria used to correlate the difficulty of surgery with the preoperative score are detailed in Table 2.

**Table 2:** Intraoperative assessment criteria for the difficulty of laparoscopic cholecystectomy

Factors	Easy	Difficult	Very Difficult
Time taken (minutes)	<60 min	60–120 min	>120 min
Bile/stone spillage	No	Yes	Yes
Injury to duct or artery	No	Duct only	Both
Conversion to open surgery	No	No	Yes

The subjective technical difficulties during surgery were reported separately from the intraoperative assessment parameters, such as difficulties encountered during cystic duct clipping, Calot's triangle dissection, umbilical port entry, gall bladder grasping, adhesiolysis, artery clipping, and extraction of the gall bladder.

Bias control and postoperative care

To avoid discrepancies in the results, all patients included in the study were operated on by the same laparoscopic surgeon, who had good blinding of the preoperative difficulty score. All enrolled patients received the same antibiotic regimen and standardized postoperative care.

Ethical approval

The study was approved by the Institutional Ethical Committee, and informed consent was obtained from all participants prior to inclusion in the study.

Statistical analysis

Statistical analysis was performed using Statistical Product and Service Solutions (SPSS, version 21.0; IBM SPSS Statistics for Windows, Armonk, NY). Categorical variables were presented as numbers and percentages (%), while continuous variables were presented as mean ± SD and median. Normality of data was assessed using the Kolmogorov-Smirnov test, and when normality was rejected, non-parametric tests were applied. Quantitative variables were compared between the two groups (easy and difficult laparoscopic cholecystectomy) using the unpaired t-test or Mann-Whitney test when the data were not normally distributed. Qualitative variables were analyzed using the chi-square test or Fisher's exact test as appropriate. Sensitivity, specificity, positive predictive value, and negative predictive value were calculated for the scoring system to predict difficult laparoscopic cholecystectomy. Univariate and multivariate logistic regression analyses were performed to identify risk factors associated with difficult laparoscopic cholecystectomy. A p-value of less than 0.05 was considered statistically significant.

## Results

Demographic and clinical characteristics

Of the 150 patients enrolled in the study, 28 (18.67%) were male, and 122 (81.33%) were female. The majority of patients (126 patients) were aged ≤50 years, while 24 patients (16%) were aged >50 years. Regarding body mass index (BMI), 57 patients (38%) had a BMI <25 kg/m², 91 patients (60.67%) had a BMI between 25.1 and 27.5 kg/m², and two patients (1.33%) had a BMI >27.5 kg/m².

A history of previous surgery was documented in 37 patients, including 35 patients (23.33%) with infraumbilical scars and two patients (1.33%) with supraumbilical scars. A prior history of hospital admission was present in 38 patients (25.33%). None of the patients had a history of jaundice. Comorbidities were observed in 20 patients, including 13 patients (8.67%) with diabetes mellitus and seven patients (4.67%) with hypertension. The gallbladder was palpable in one patient (0.67%).

Ultrasonographic findings

On ultrasonography, gallbladder wall thickness was normal in 131 patients (87.33%) and increased in 19 patients (12.67%). An impacted gallstone was identified in one patient (0.67%), and a pericholecystic collection was observed in five patients (3.33%).

Intraoperative findings and complications

Intraoperative assessment revealed bile spillage in 31 patients (20.67%), stone spillage in 13 patients (8.67%), cystic artery injury in three patients (2.0%), bile duct injury in one patient (0.67%), and conversion to open cholecystectomy in five patients (3.33%). The duration of surgery was <60 minutes in 120 patients (80.0%), 60-120 minutes in 27 patients (18%), and >120 minutes in three patients (2.0%).

Distribution of preoperative scores

Based on the preoperative scoring system, 108 patients (72%) were categorized as easy, 37 patients (24.67%) as difficult, and five patients (3.33%) as very difficult laparoscopic cholecystectomy.

Correlation of preoperative factors with operative difficulty

Postoperative outcomes were correlated with the parameters included in the scoring system, and statistical analysis was performed to assess the significance of each factor (Table 3). A prior history of hypertension, previous hospitalisations, increased gallbladder wall thickness, and the presence of pericholecystic collection were found to be significantly associated with increased difficulty during laparoscopic cholecystectomy.

**Table 3:** Comparison of the association of the preoperative variables and preoperative score with easy and difficult laparoscopic cholecystectomy with their respective P values and statistical test used BMI: Body mass index; GB: Gall bladder; SD: Standard deviation; Statistical test used: Fisher's exact test, chi-square test, and Mann–Whitney test

Parameter	Easy	Difficult	p-value, tests	Interpretation
Age (mean ± SD)	37.44 ± 12.39	40.98 ± 12.56	0.121, Fisher's exact test	Not significant
Gender: Female	89 (72.95%)	33 (27.05%)	0.588, Chi-square test	Not significant
Gender: Male	19 (67.86%)	9 (32.14%)		Not significant
Diabetes: No	101 (73.72%)	36 (26.28%)	0.127, Chi-square test	Not significant
Diabetes: Yes	7 (53.85%)	6 (46.15%)		Not significant
Hypertension: No	107 (74.83%)	36 (25.17%)	0.002, Fisher's exact test	Significant
Hypertension: Yes	1 (14.29%)	6 (85.71%)		Significant
History of hospitalization: No	86 (76.79%)	26 (23.21%)	0.025, Chi-square test	Significant
History of hospitalization: Yes	22 (57.89%)	16 (42.11%)		Significant
BMI: <25	45 (78.95%)	12 (21.05%)	0.224, Fisher's exact test	Not significant
BMI: 25–27.5	61 (67.03%)	30 (32.97%)		Not significant
BMI: ≥27.5	2 (100%)	0 (0%)		Not significant
Abdominal scar: No	83 (73.45%)	30 (26.55%)	0.112, Fisher's exact test	Not significant
Abdominal scar: Infraumbilical	25 (71.43%)	10 (28.57%)		Not significant
Abdominal scar: Supraumbilical	0 (0%)	2 (100%)		Significant
Palpable GB: No	108 (72.48%)	41 (27.52%)	0.28, Fisher's exact test	Not significant
Palpable GB: Yes	0 (0%)	1 (100%)		Not significant
GB wall thickness: < 4 mm	103 (78.63%)	28 (21.37%)	<0.0001, Chi-square test	Significant
GB wall thickness: > 4 mm	5 (26.32%)	14 (73.68%)		Significant
Pericholecystic collection: No	108 (74.48%)	37 (25.52%)	0.001, Fisher's exact test	Significant
Pericholecystic collection: Yes	0 (0%)	5 (100%)		Significant
Impacted stone: No	108 (72.48%)	41 (27.52%)	0.28, Fisher's exact test	Not significant
Impacted stone: Yes	0 (0%)	1 (100%)		Not significant
Preoperative score (mean ± SD)	2.06 ± 2.01	3.81 ± 3.29	0.006, Mann–Whitney	Significant

Association of various intraoperative parameters with easy and difficult surgeries has been depicted in Table 4.

**Table 4:** Comparison of association of the intraoperative variables with easy and difficult laparoscopic cholecystectomy with their respective P values and statistical test used Fisher's exact test - statistical test used in analysis

Parameter	Easy	Difficult	p-value, tests	Interpretation
Bile spillage: No	108 (90.76%)	11 (9.24%)	<0.0001, Fisher's exact test	Significant
Bile spillage: Yes	0 (0%)	31 (100%)		Significant
Stone spillage: No	108 (78.83%)	29 (21.17%)	<0.0001, Fisher's exact test	Significant
Stone spillage: Yes	0 (0%)	13 (100%)		Significant
Bile duct/artery injury: No	108 (73.97%)	38 (26.03%)	0.006, Fisher's exact test	Significant
Artery injury	0 (0%)	3 (100%)		Significant
Bile duct injury	0 (0%)	1 (100%)		Significant
Surgery time: <60 min	108 (90.0%)	12 (10.0%)	<0.0001, Fisher's exact test	Significant
Surgery time: 60–120 min	0 (0%)	27 (100%)		Significant
Surgery time: >120 min	0 (0%)	3 (100%)		Significant
Conversion to open	0 (0%)	5 (100%)	—	Significant

All the variables that were included in the logistic regression model and the adjusted odds ratios with 95% confidence intervals are depicted in Table 5.

**Table 5:** Multivariate logistic regression to assess the independent significant predictors of difficult laparoscopic cholecystectomy GB: Gall bladder

Variables	Beta coefficient	Standard error	P value	Adjusted odds ratio	Adjusted odds ratio lower bound (95%)	Adjusted odds ratio upper bound (95%)
Preoperative score	-0.277	0.559	0.620	0.758	0.254	2.265
Hypertension	0.665	2.145	0.757	1.944	0.029	130.230
History of hospitalization	0.987	2.556	0.699	2.682	0.018	401.953
GB wall thickness						
<4mm				1	0.064	
>4mm	1.102	1.963	0.574	3.010		140.957
Pericholecystic collection	-0.027	2.246	0.990	0.973	0.012	79.411
Bile spillage	5.096	1.200	<0.0001	163.440	15.565	1716.145
Stone spillage	-0.431	1.675	0.797	0.650	0.024	17.309
Bile duct/artery injury						
No				1		
Artery injury	5.025	2.320	0.030	152.212	1.613	14366.338
Bile duct injury	8.194	106.918	0.939	3618.9	3.54903E-88	3.69015E+94
Surgery time(minutes)						
<60 minutes				1		
60 to 120 minutes	4.853	1.273	0.0001	128.149	10.562	1554.867
>120 minutes	-0.705	2.757	0.798	0.494	0.002	109.791

Specific intraoperative technical difficulties

**Figure 1:**
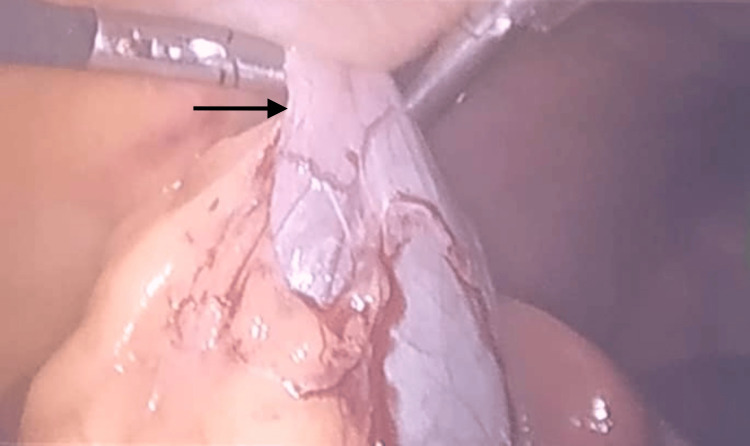
Omentum adhered with the gall bladder

**Figure 2:**
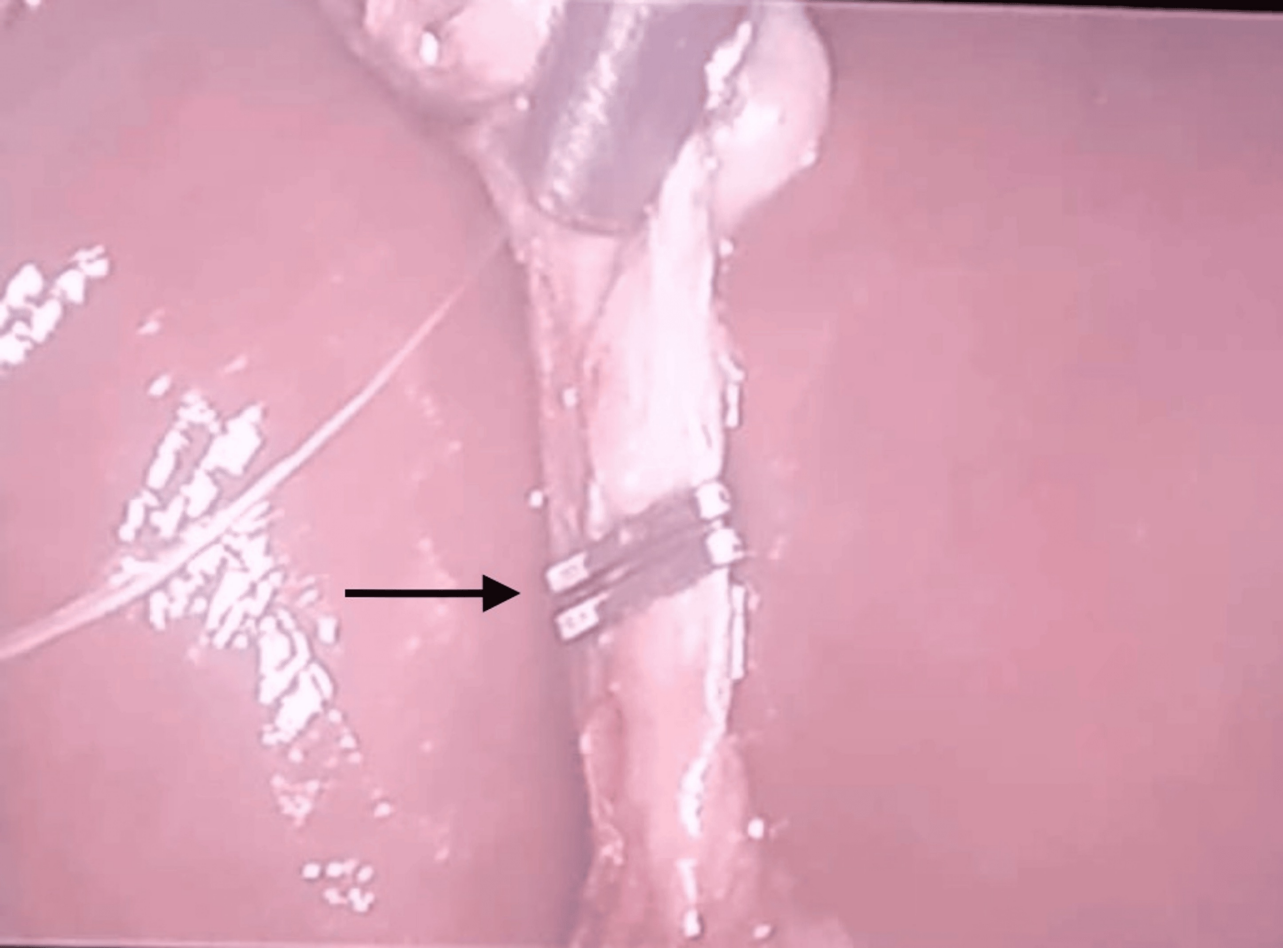
Difficult clipping of the cystic duct

Adhesion of the omentum to the gallbladder was observed in one case, and difficulty in clipping of the cystic duct was encountered in one case, as shown in Figures 1-2.

Other technical difficulties encountered during surgery were at umbilical port entry in 6.6% cases, at adhesiolysis in 12% cases, at Calot’s dissection in 14.6% cases, at artery clipping in 3.3% cases, and at extraction of gall bladder in 0.66%.

Diagnostic performance of the preoperative scoring system

For ROC curve analysis and binary outcome assessment, operative difficulty was dichotomized. Cases classified as easy were considered non-difficult, while difficult and very difficult cases were combined and analyzed as a single difficult group. This approach was necessary because receiver operating characteristic (ROC) analysis requires a binary outcome, and the number of very difficult cases was too small for reliable independent analysis.

ROC curve analysis demonstrated that the discriminatory power of the preoperative score was acceptable, with an area under the curve (AUC) of 0.749 (95% CI: 0.672-0.816). A preoperative score >3 was identified as the optimal cutoff for predicting difficult laparoscopic cholecystectomy, with an AUC of 0.749. The duration of surgery showed a significant association with operative difficulty.

**Figure 3:**
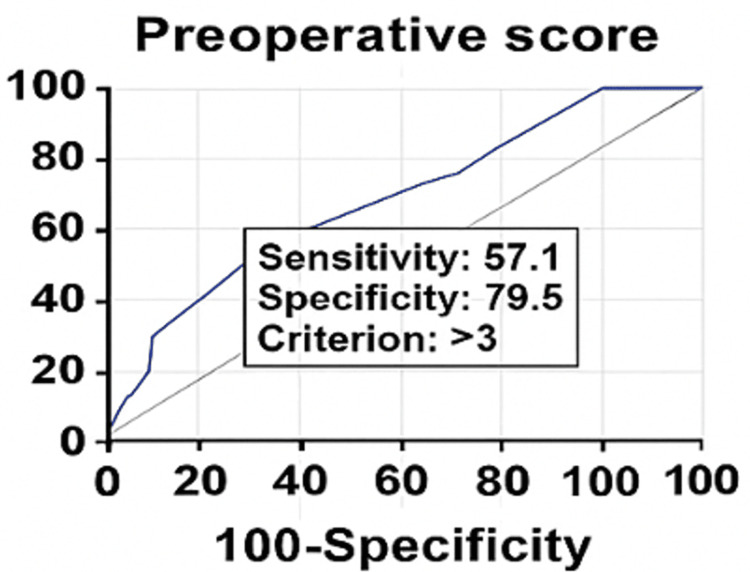
ROC curve of the preoperative scores for difficult LC LC: Laparoscopic cholecystectomy; ROC: Receiver operating characteristic; PPV: Positive predictive value; NPV: Negative predictive value; CI: Confidence interval; AUC: Area under the ROC curve

As shown in Table 6 and Figure 3, among patients who underwent difficult laparoscopic cholecystectomy, 57.14% had a preoperative score >3. A preoperative score >3 was associated with a 39.00% probability of a difficult laparoscopic cholecystectomy, whereas a score ≤3 was associated with an 11.00% probability. Among patients who underwent easy laparoscopic cholecystectomy, 79.51% had a preoperative score ≤3. Sensitivity of the score was 57.14%, specificity was 79.51%, positive predictive value was 39%, negative predictive value was 85%, and the diagnostic accuracy of the score was 75.35%.

**Table 6:** Sensitivity, specificity, PPV, NPV, AUC, and P values ROC: Receiver operating characteristic; PPV: Positive predictive value; NPV: Negative predictive value; CI: Confidence interval; AUC: Area under the ROC curve

Variables	Values
AUC	0.749
Standard error	0.0539
95% CI	0.672-8.16
P value	<0.001
Sensitivity (95% CI)	57.14% (37.2-75.5%)
Specificity (95% CI)	79.51% (71.3-86.3%)
PPV (95% CI)	39% (24.2-55.5%)
NPV (96% CI)	85% (81.6-94.2%)
Diagnostic accuracy	75.35%

**Figure 4:**
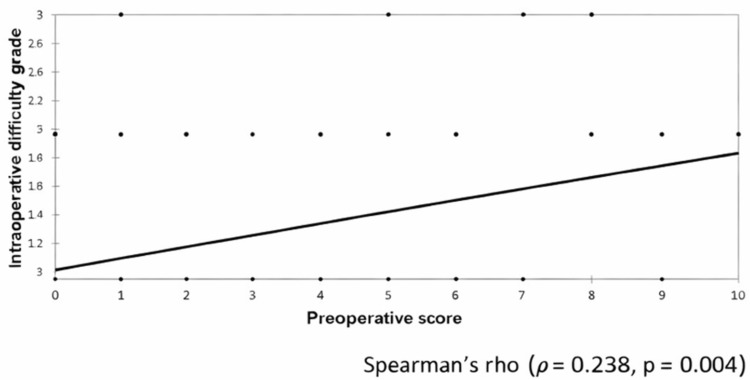
Correlation of the preoperative score with the intraoperative difficulty grade Spearman's rho: Spearman's rank correlation coefficient

Significant positive correlation was seen between the preoperative score and the intraoperative difficulty grade, with a correlation coefficient of 0.238, as shown in Table 7 and Figure 4.

**Table 7:** Variables relevant to correlating preoperative score with intraoperative difficulty

Variables (preoperative score)	Intraoperative difficulty grade
Correlation coefficient	0.238
P value	0.004

## Discussion

Overview of the study

Laparoscopic cholecystectomy is among the most frequently performed surgical procedures worldwide and continues to undergo periodic advancements with evolving technology to enhance safety, cosmetic outcomes, and cost-effectiveness. The present study aimed to evaluate various preoperative predictors, including historical, clinical, and imaging parameters. A study involving 150 patients to assess preoperative predictors of difficult laparoscopic cholecystectomy yielded the following observations.

Gender and operative difficulty

Gender distribution did not show a statistically significant association with operative difficulty in this study: female (27.05%) vs male (32.14%) (p = 0.588). Although male gender was associated with difficult surgeries in some series, the present findings suggest that gender alone does not reliably predict operative complexity.

Hypertension as a predictor

Hypertension was found to be significantly associated with difficult laparoscopic cholecystectomy in this study, with a p value of 0.002. A substantially higher proportion of hypertensive patients experienced operative difficulty compared to non-hypertensive patients. This association has also been reported in previous studies, suggesting that comorbid conditions may contribute indirectly to operative challenges. Similar findings have been seen in a study by Dhanke et al. [10].

Previous hospitalisation and operative difficulty

A prior history of hospitalization for gallbladder-related illness showed a significant association with difficult laparoscopic cholecystectomy. This may be attributed to repeated inflammatory episodes, leading to fibrosis, adhesions, and distorted anatomy, thereby increasing technical difficulty during dissection. Similar findings have been documented in earlier studies performed by Ahmed et al. [11] and Bhandari et al. [12].

Ultrasonographic predictors

Ultrasonographic parameters played a crucial role in predicting operative difficulty. Gallbladder wall thickness greater than 4 mm demonstrated a strong and statistically significant association with difficult laparoscopic cholecystectomy, with a p value of <0.0001, consistent with previously published literature. Thickened gallbladder walls are indicative of chronic inflammation and fibrosis, which complicate grasping and dissection and creating proper critical view of safety. Similar findings were seen in a study performed by Joshi et al. [13], Khetan et al. [14], and Ramírez-Giraldo et al. [15].

The presence of pericholecystic collection was also significantly associated with higher preoperative scores and increased operative difficulty. This finding reflects ongoing or previous inflammatory processes, which are known to increase the likelihood of adhesions and distorted anatomy.

Intraoperative complications and preoperative score

Intraoperative complications, such as bile spillage, stone spillage, bile duct injury, and cystic artery injury, were exclusively observed in difficult laparoscopic cholecystectomy cases, with a p value <0.0001, showing a strong association with higher preoperative scores. These findings underscore the clinical relevance of accurate preoperative risk stratification, in line with the findings seen in a study by Randhawa and Pujahari [8].

Operative time

Operative time was significantly longer in difficult cases, with all such procedures exceeding 60 minutes, with a p value of <0.0001. This observation further validates the use of operative duration as an indirect indicator of surgical complexity.

Preoperative score and intraoperative difficulty

There was a significant difference between the preoperative score and conversion to open surgery, adhesiolysis, and Calot's dissection. Conversion to open surgery was associated with the preoperative score, with a p value of 0.02; adhesiolysis was associated with the preoperative score, with a p value of 0.04; and Calot's triangle dissection was associated with the preoperative score, with a p value of 0.009. Intraoperative difficulties had an association with the preoperative score in the studies done by Vivek et al. [16] and Reynolds [17].

Technical difficulties during surgery

Difficulty was reported by surgeons at umbilical port entry in 6.6% cases, at gall bladder grasping in 2.6% cases, at adhesiolysis in 12% cases, at Cabot's dissection in 14.6% cases, at duct clipping in 4.6% cases, at artery clipping in 3.3% cases, and at extraction of gall bladder in 0.66%. However, no significant association was seen in the preoperative score with umbilical port entry, GB grasping, duct clipping, artery clipping, and extraction of the gall bladder. Findings were unlike those found in a study done by Vivek et al. [16] and Ghnnam et al. [18].

Age and operative difficulty

Most patients belonged to the 31-40 years age group (35.33%, n = 53). The number of patients above 60 years of age is 10 (667%). The mean patient age is 38.43+/-12.5, with a median of 36. Age >50 years was found to be more associated with difficult surgery, but the association was not significant. It was identified as a significant predictor for preoperative assessment of laparoscopic cholecystectomy in the studies done by Kumar et al. [19], Kumar et al. [20], HamdyShaban et al. [21].

Effectiveness of the scoring system

The scoring system applied in the present study effectively predicts the difficulty of laparoscopic cholecystectomy, demonstrating high sensitivity and specificity. The diagnostic accuracy of the current scoring system is also high, with a significant association. Findings were similar in the study done by Randhawa and Pujahari in 2009, in which the preoperative scoring proved to be both statistically and clinically effective in predicting operative outcomes in laparoscopic cholecystectomy [8].

Clinical implications

Overall, the findings support the usefulness of a simple, preoperative scoring system in anticipating difficult laparoscopic cholecystectomy. Such predictive tools can enhance surgical preparedness, improve patient safety, reduce unexpected intraoperative challenges, prepare for referral to a hepatobiliary surgeon, and optimise healthcare resource utilisation.

Study limitations and future directions

Although the scoring system demonstrated high sensitivity and specificity, the limited sample size may constrain the generalizability of these findings. Statistical power and confidence interval precision depend on both sample size and event distribution. Larger sample sizes increase power and yield narrower, more precise confidence intervals, whereas small samples reduce power and widen intervals. Similarly, low or unevenly distributed events decrease the effective sample size, leading to reduced power and unstable, wide confidence intervals, even in studies with large overall cohorts. Hence, larger multicentre studies are warranted to further validate the scoring system and enhance the predictive accuracy of its individual components.

## Conclusions

A preoperative scoring system incorporating simple clinical and ultrasonographic parameters can effectively predict the difficulty of laparoscopic cholecystectomy. The application of this scoring system provides a reliable and practical tool for preoperative assessment, enabling surgeons to anticipate operative challenges, improve surgical planning, optimize resource allocation, and enhance patient counselling. Its use may contribute to improved operative outcomes and procedural safety, particularly in settings where early identification of difficult laparoscopic cholecystectomy is essential.

It should be incorporated into routine preoperative evaluation by preparing a checklist to enhance surgical outcomes and reduce intraoperative surprises.
